# Protective Effects of Astaxanthin on ConA-Induced Autoimmune Hepatitis by the JNK/p-JNK Pathway-Mediated Inhibition of Autophagy and Apoptosis

**DOI:** 10.1371/journal.pone.0120440

**Published:** 2015-03-11

**Authors:** Jingjing Li, Yujing Xia, Tong Liu, Junshan Wang, Weiqi Dai, Fan Wang, Yuanyuan Zheng, Kan Chen, Sainan Li, Huerxidan Abudumijiti, Zheng Zhou, Jianrong Wang, Wenxia Lu, Rong Zhu, Jing Yang, Huawei Zhang, Qin Yin, Chengfen Wang, Yuqing Zhou, Jie Lu, Yingqun Zhou, Chuanyong Guo

**Affiliations:** 1 Department of Gastroenterology, Shanghai Tenth People’s Hospital, Tongji University School of Medicine, Shanghai, 200072, China; 2 Department of Gastroenterology, Shanghai Tenth People’s Hospital, The First Clinical Medical College of Nanjing Medical University, Nanjing, 210029, China; 3 Department of Gastroenterology, Shanghai Tenth People’s Hospital, Soochow University, Suzhou, 215006, China; Institute of Hepatology - Birkbeck, University of London, UNITED KINGDOM

## Abstract

**Objective:**

Astaxanthin, a potent antioxidant, exhibits a wide range of biological activities, including antioxidant, atherosclerosis and antitumor activities. However, its effect on concanavalin A (ConA)-induced autoimmune hepatitis remains unclear. The aim of this study was to investigate the protective effects of astaxanthin on ConA-induced hepatitis in mice, and to elucidate the mechanisms of regulation.

**Materials and Methods:**

Autoimmune hepatitis was induced in in Balb/C mice using ConA (25 mg/kg), and astaxanthin was orally administered daily at two doses (20 mg/kg and 40 mg/kg) for 14 days before ConA injection. Levels of serum liver enzymes and the histopathology of inflammatory cytokines and other maker proteins were determined at three time points (2, 8 and 24 h). Primary hepatocytes were pretreated with astaxanthin (80 μM) in vitro 24 h before stimulation with TNF-α (10 ng/ml). The apoptosis rate and related protein expression were determined 24 h after the administration of TNF-α.

**Results:**

Astaxanthin attenuated serum liver enzymes and pathological damage by reducing the release of inflammatory factors. It performed anti-apoptotic effects via the descending phosphorylation of Bcl-2 through the down-regulation of the JNK/p-JNK pathway.

**Conclusion:**

This research firstly expounded that astaxanthin reduced immune liver injury in ConA-induced autoimmune hepatitis. The mode of action appears to be downregulation of JNK/p-JNK-mediated apoptosis and autophagy.

## Introduction

The liver, the largest digestive gland, is the center of energy metabolism in the body. Hepatitis is a condition characterized by inflammation of the liver and the presence of inflammatory cells in the liver tissue. Autoimmune hepatitis is a chronic disease caused by an abnormal immune response against liver cells. The incidence of severe autoimmune hepatitis that develops into liver cirrhosis, liver failure or even death has dramatically increased in Europe, the United States and Asian countries in recent times [1,2]. At present, the etiology of this chronic disease is not fully understood [3]. Currently, this condition is therapeutically controlled by administration of glucocorticoid combined with azathioprine, however, side effects are experienced due to impaired immunity and a disturbed endocrine system [4]. The identification of effective and safe treatment options for autoimmune hepatitis is therefore urgently required.

Effective drug screening programs for hepatitis depend on the establishment of suitable animal models able to closely resemble the pathological process that occurs in the human liver. Many models of drug-induced liver injury mimic the development of various types of hepatitis, including those established with bacillus Chalmette—Guerin (BCG)/lipopolysaccharide (LPS), d-galactosamine (d-GalN)/LPS, or CCl_4_. The mechanism by which they induce liver injury partly depend on the activation of T cells and macrophages to produce inflammatory cytokines, such as TNF-α, IL-6, IL-1β, and IFN-γ [5,6]. Among these models, ConA-induced liver injury is popular because it is dose dependent and simple to establish. In 1992, Tiegs and colleagues successfully established a concanavalin A (ConA)-induced immunological liver injury mouse model [7]. ConA is a plant blood lectin that promotes cell division. ConA has been shown to strongly activate intrahepatic CD4+ T cells and macrophages that entered into the hepatic sinus causing proliferation and the production of cytokines, including TNF-α, IL-6, IL-1β and IFN-γ [8–11], directly or indirectly leading to liver damage. In addition, nuclear transfer of nuclear factor-κB p65 (NF-κB p65) and the interaction of ICAM-1/LFA-1 between lymphocytes and hepatocytes also played a role in liver cell damage [12,13]. Research has shown that cytokine production peaked before lymphocyte infiltration indicating the association between high cytokine levels and early liver damage [14]. TNF-α was the dominant cytokine causing irreversible, detrimental biological effects in many types of drug-induced liver injury, including those induced with ConA, BCG/LPS, or d-GalN/LPS [15–17].

Previous studies have demonstrated that the pathogenesis of liver injury caused by ConA-induced autoimmune hepatitis involved apoptosis and autophagy [18–20]. Apoptosis, first defined by Kerr and colleagues, is a biochemical and morphological process triggered by extrinsic and intrinsic pathways that both activate cysteine proteases known as caspases [21]. As the major effector in ConA damage, blockage of TNF-α synthesis had anti-inflammatory and anti-apoptotic effects [22]. Serum TNF-α interacted with the death domain of the adapter molecule TNF receptor-associated protein (TRADD) through activating TNF receptor 1 (TNFR1) combined with TNF receptor-associated factor 2 (TRAF2), leading to the formation of the signal transducer Fas-associated protein with death domain (FADD) and apoptosis [23]. The Bcl-2 protein family, which is representative of the intrinsic pathway, was involved in the regulation process. Autophagy, first described by Ashford and Porter, is a catabolic process accompanied by the formation of autophagosomes and autolysosomes, which leads to the massive degradation of organelles such as the mitochondria and endoplasmic reticulum [24]. As a peculiar phenomenon of eukaryotic cells, autophagy is a doubled-edged sword, facilitating either cell survival or death. Increasing evidence suggests that autophagy negatively regulates the liver protection mechanism [25,26]. However, in the animal model of ConA-induced hepatitis, TNF-α could participate in autophagy through the interactions between Beclin-1 and Bcl-2 or between FADD and Atg5 [27].

Astaxanthin (3, 3'-dihydroxy-β, β'-carotene-4, 4'-dione), a kind of carotenoid pigment naturally produced by algae, bacteria and phytoplankton, contains conjugated double bonds, hydroxyl and ketone groups involved in electron transfer and free radicals [28]. In recent years, astaxanthin has been shown to exhibit a wide range of biological effects, such as antioxidant, atherosclerosis and antitumor properties [29–32]. Recent evidence showed that astaxanthin is a potential antioxidant that plays a role in terminating the inflammatory response. Bhuvaneswari and colleagues found that astaxanthin could suppress NF-κB p65-mediated inflammation in high fructose and high fat diet-fed mice [33]. Astaxanthin was illuminated as a cardioprotective supplement through its anti-inflammatory properties, described by Nakao and colleagues [34]. Kuzuhiro and colleagues also demonstrated the effects of astaxanthin on LPS-induced inflammation in vitro and in vivo [35]. In addition, astaxanthin was shown to play an important role in protecting eyes from inflammatory infiltration and reducing inflammatory proliferation of skin [36–38]. On the one hand, astaxanthin showed clear inhibition of inflammatory cytokines such as TNF-α, IL-6, IL-1β and IFN-γ [39,40]. In ConA-induced liver injury, the damaged tissue contributed to the release of reactive oxygen species (ROS) and nitric oxide (NO). Then, astaxanthin could provide protection against hepatitis by reducing the production of ROS and NO and reducing the activity of inducible nitric oxide synthase to inhibit cyclooxygenase (COX) and TNF-α levels [35,41]. However, on the other hand, astaxanthin could also down-regulate activation and migration of NF-κB p65 mediated by ConA to attenuate the expression of NF-κB p65 in the nucleus to achieve anti-inflammatory effects [42]. Currently, the mechanism of action of astaxanthin in ConA-induced autoimmune hepatitis is unclear. However, the establishment of a ConA-induced immunological liver injury mouse model that closely resembles the pathogenic process in the human liver now provides the opportunity to study the pharmacological properties of potential hepatitis drug candidates.

In this study, we investigated the mechanism of action of astaxanthin in ConA-induced autoimmune hepatitis. We hypothesized that astaxanthin could inhibit the rise in TNF-α levels caused by ConA-induced hepatitis, in turn reducing liver damage. We also investigated the mechanism of action of astaxanthin.

## Materials and Methods

### 2.1 Reagents

Astaxanthin, ConA, and dimethyl sulfoxide (DMSO) were purchased from Sigma—Aldrich (St. Louis, MO, USA). TNF-α was purchased from Peprotech (Rocky Hill, NJ, USA). Antibodies were from Cell Signaling Technology (Danvers, MA, USA), including the antibodies against NF-κB p65, IL-6, IL-1β, IFN-γ, LC3, Beclin1, Bcl-2, Bax, JNK, p-JNK, ERK, p-ERK, P38 MAPK, p-P38 MAPK, TNF-α, and TRAF2. The alanine aminotransferase (ALT) and aspartate aminotransferase (AST) microplate test kits were purchased from Nanjing Jiancheng Bioengineering Institute (Jiancheng Biotech, China). The RNA polymerase chain reaction (PCR) kit was purchased from Takara (Takara Biotechnology, Dalian, China). The cell counting kit (CCK8) was produced by Dojindo (Dojindo Laboratories, Japan). The Annexin V-APC/7-AAD apoptosis detection kitwas purchased from BD Biosciences (San Jose, CA, USA).

### 2.2 Animals

Male Balb/c mice weighing between 20 and 25 g (7–9 weeks old) were purchased from Shanghai Laboratory Animal Co. Ltd. (SLAC, Shanghai, China). The mice were housed in a clean room at a temperature of 23±2°C and a humidity of 50% with a 12 h alternating light and dark cycle. They were permitted free access to food and water. All animal experiments were performed according to the National Institutes of Health Guidelines for the Care and Use of Laboratory Animals and were approved by the Animal Care and Use Committee of Shanghai Tongji University, China.

### 2.3 Hepatocyte isolation

Primary hepatocytes were isolated with a two-step perfusion method [43]. Briefly, the executed mice were laparotomized after soaking in 75% ethanol. The hepatic portal vein was perfused with 10 mL of prewarmed d-Hanks buffer for 10 min, and then with 5 ml of 0.02% type V collagenase solution. The removed liver tissues were cut into small pieces and placed in collagenase V solution in a shaking water bath for approximately 30 min. The cell suspensions were then filtered into a glass tube and centrifuged at 800 g for 5 min. RPMI-1640 culture medium was added to the washed primary hepatocytes, which were then incubated at 37°C under 5% CO_2_. The viability of the isolated hepatocytes was determined with Trypan blue exclusion, and exceeded 95%.

### 2.4 Cell culture and CCK8 assay

The primary hepatocytes were cultured in RPMI-1640 culture medium (Thermo, China) supplemented with 10% fetal bovine serum (Hyclone, South America), 100 U/mL penicillin, and 100 g /ml streptomycin (Gibco, Canada) in a humidified incubator at 37°C under 5% CO_2_. The cells were plated at a density 2 10^4^ cells/well in 96-well plates (100 μL medium per well). The concentration of TNF-α was 10 ng/ml and the astaxanthin concentration was 20, 40, 60, 80, 100, or 120 M. Cell viability was measured with the CCK8 assay at a wavelength of 450 nm.

The primary hepatocytes were divided into five groups:

1. Control group: no treatment;
2. Astaxanthin group: treated with astaxanthin diluted in DMSO at a concentration of 80 μM;
3. DMSO group: treated with DMSO at a concentration of 80 μM;
4. TNF-α group: treated with TNF-α dissolved at a concentration of 20 ng/ml;
5. TNF-α+astaxanthin group: astaxanthin administered 24 h before stimulation with TNF-α.

### 2.5 Preliminary study

A total of 72 mice were randomly divided into four groups: group A was given no treatment and group B was lavaged with olive oil. Astaxanthin was dissolved in olive oil and orally administered at a daily dose of 20 mg/kg (group C) or 40 mg/kg (group D) for two weeks. Six mice randomly selected from the four groups were killed. Sera and liver tissues were collected and analyzed for liver enzymes, immune cell subsets, cytokine levels and pathological changes.

### 2.6 Drug preparation

ConA was dissolved in pyrogen-free saline at a concentration of 2.5 mg/ml and injected at a dose of 25 mg/kg body weight to induce hepatitis as previously described [11]. All 96 mice were treated by tail intravenous injection of ConA 1 h before drawing materials. The mice were randomly divided into four groups, as follows:

1. Normal group (n = 24): lavage for olive oil;
2. ConA group (n = 24): ConA injected via tail vein after lavage with olive oil;
3. Low dose group (n = 24): ConA + 20 mg/(kg·d) astaxanthin;
4. High dose group (n = 24): ConA + 40 mg/(kg·d) astaxanthin.

### 2.7 Serum liver enzyme analysis and cytokine assessment

Blood obtained from individual mice was collected at 2, 8 and 24 h after ConA induction, according to a previous study [44]. After 5 h standing, the serum was separated by centrifugation at 4300 g for 10 min at 4°C and used in the detection of liver function and cytokine levels. ALT and AST were measured using an automated chemical analyzer (Olympus AU1000, Japan) and NF-κB p65 and IL-6 levels were assessed using enzyme-linked immunosorbent assay (ELISA) kits (R&D Systems, USA) according to the manufacturers’ protocols.

### 2.8 Histopathology

A portion of the live tissue from individual mice that had been fixed in 4% paraformaldehyde was subjected to dehydration and penetration. The specimen was then embedded in paraffin. The section was cut at a thickness of 5 μm for hematoxylin and eosin (H&E) staining. Any changes in liver pathology were assessed by light microscopy.

### 2.9 Immunohistochemistry

Prepared paraffin sections that had been baked for 1 h at 60°C were dewaxed and rehydrated using xylene and different concentrations of alcohol. Antigens immersed in the citrate buffer were recovered by the heat-induced antigen retrieval technique, which involved heating in a water bath at 95°C for 10 min, then cooling to room temperature. Hydrogen peroxide solution (3%) was added to the specimens that were then stored for 20 min at 37°C to block endogenous peroxidase activity. The sections were then washed with phosphate buffer solution (PBS) three times and blocked with 5% bovine serum albumin (BSA) at 37°C for 20 min, followed by a 10-min incubation at room temperature. Next, the liver specimens were incubated overnight at 4°C with primary antibodies including anti-LC3 I/II (diluted 1:500), anti-Beclin-1 (diluted 1:500), anti-p-JNK (diluted 1:100) and anti-TNF-α (diluted 1:100). On the second day, the slices stained using a diaminobenzidine (DAB) kit were slide-integrated and then observed by light microscopy. Color development was filmed using a digital camera (Olympus) mounted on a microscope (Leica, Wetzlar, Germany). The integrated optical densities (IOD) of different indicators were calculated using Image-Pro Plus software 6.0 (Media Cybernetics, Silver Spring, MD, USA).

### 2.10 Western blotting analysis

Total protein was extracted using radio immunoprecipitation assay (RIPA) lysis buffer with protease inhibitors (PI) and phenylmethane-sulfonyl fluoride (PMSF) from the liver tissue stored at −80°C. The concentration of the prepared protein was calculated using the bicinchoninic acid (BCA) protein assay (Kaiji, China) and samples were prepared in 5× sodium dodecyl sulfate-polyacrylamide gel electrophoresis (SDS—PAGE) sample loading buffer. The proteins were separated on 8%–12.5% SDS-polyacrylamide gels and transferred onto polyvinylidene fluoride (PVDF) membranes. PBS containing 0.1% Tween 20 (PBST) and 5% non-fat dried milk was used to block non-specific binding sites, then membranes were incubated overnight at 4°C with primary antibodies: β-actin (1:1000), NF-κB p65 (1:500), IL-6 (1:500), IL-1β (1:200), IFN-γ (1:200), LC3 (1:1000), Beclin1 (1:500), Bcl-2 (1:1000), Bax (1:500), JNK (1:1000), p-JNK (1:500), ERK(1:500), p-ERK(1:500), P38 MAPK (1:1000), p-P38 MAPK (1:500), TNF-α (1:500) and TRAF2 (1:1000). Membranes were then washed with PBST three times and incubated with the secondary antibody horseradish peroxidase-conjugated anti-rabbit or anti-mouse IgG (1:2000) for 1 h at room temperature. Finally, the membranes were washed three times and scanned using the Odyssey two-color infrared laser imaging system (fluorescence detection). Molecular sizes were determined by comparison with the prestained molecular weight markers.

### 2.11 Reverse transcription (RT)-PCR and quantitative real time (qRT)-PCR

We extracted the total RNA from frozen liver tissues and then transcribed it into cDNA using the reverse transcription kit (TaKaRa Biotechnology, China), as instructed by the manufacturer. SYBR Green quantitative RT-PCR was performed to determine the gene expression level using a 7900HT fast real-time PCR system (Applied Biosystems, CA, USA), according to the protocols provided with the SYBR Premix EX Taq (TaKaRa Biotechnology, China). The levels of target gene were normalized with respect to the data for the β-actin gene. The primer sequences used in the experiment are shown in Table 1.

**Table 1:** Nucleotide sequences of primers used for qRT-PCR.

Gene		Primer sequence (5'—3')
NF-κB p65	Forward	ATGGCAGACGATGATCCCTAC
	Reverse	CGGATCGAAATCCCCTCTGTT
IL-6	Forward	CTGCAAGAGACTTCCATCCAG
	Reverse	AGTGGTATAGACAGGTCTGTTGG
IL-1β	Forward	CGATCGCGCAGGGGCTGGGCGG
	Reverse	AGGAACTGACGGTACTGATGGA
IFN-γ	Forward	GCCACGGCACAGTCATTGA
	Reverse	TGCTGATGGCCTGATTGTCTT
LC3	Forward	GACCGCTGTAAGGAGGTGC
	Reverse	AGAAGCCGAAGGTTTCTTGGG
Beclin1	Forward	ATGGAGGGGTCTAAGGCGTC
	Reverse	TGGGCTGTGGTAAGTAATGGA
Bax	Forward	AGACAGGGGCCTTTTTGCTAC
	Reverse	AATTCGCCGGAGACACTCG
Bcl-2	Forward	GCTACCGTCGTCGTGACTTCGC
	Reverse	CCCCACCGAACTCAAAGAAGG
TNF-α	Forward	CAGGCGGTGCCTATGTCTC
	Reverse	CGATCACCCCGAAGTTCAGTAG
TRAF2	Forward	AGAGAGTAGTTCGGCCTTTC
	Reverse	GTGCATCCATCATTGGGACAG
β-actin	Forward	GGCTGTATTCCCCTCCATCG
	Reverse	CCAGTTGGTAACAATGCCATGT

### 2.12 Transmission electron microscopy (TEM)

The flushed liver tissue was perfused with 2% glutaraldehyde buffered with 0.2 mmol/L cacodylate and postfixed in osmium tetroxide (OsO_4_). Then the sections were viewed by electron microscopy (JEM1230, JEOL, Japan) and the images were printed onto photographic paper.

### 2.13 Detection of apoptosis and immune cell subsets with flow cytometry

Primary hepatocytes were plated in 12-well plates. Cells in the control group, astaxanthin group, DMSO group, TNF-α group, and TNF-α+astaxanthin group were collected after 24 h. After the cells were washed twice with cold PBS, suspended in 1 binding buffer, and then incubated for 15 min with 5 μL of annexin-V/APC. 7-Amino-actinomycin D (7-AAD) (5 μL) and another 200 μL of binding buffer were added before the machine-readable measurements were made.

The monoplast suspension of liver tissue were stained with phycoerythrin-conjugated CD3, CD4, CD8, CD16+56 and CD19 antibodies (Miltenyi Biotec, Auburn, CA) and incubated for 30 min. The labeled cells were analyzed by the flow cytometry in accordance with the manufacturer’s protocols.

### 2.14 Statistical analysis

The experimental data were evaluated by calculating the mean±SD. Student’s t test and one-way analysis of variance (ANOVA), followed by the Tukey’s test when F was significant, were performed to compare the differences between the experimental groups according to their characteristics. Statistical significance was assumed at P<0.05. All statistical analyses were calculated using the GraphPad Prism Software version 6.0 for Windows (GraphPad, San Diego, USA).

## Results

### 3.1 Olive oil and astaxanthin do not affect liver function or the inflammatory response

**Fig 1:**
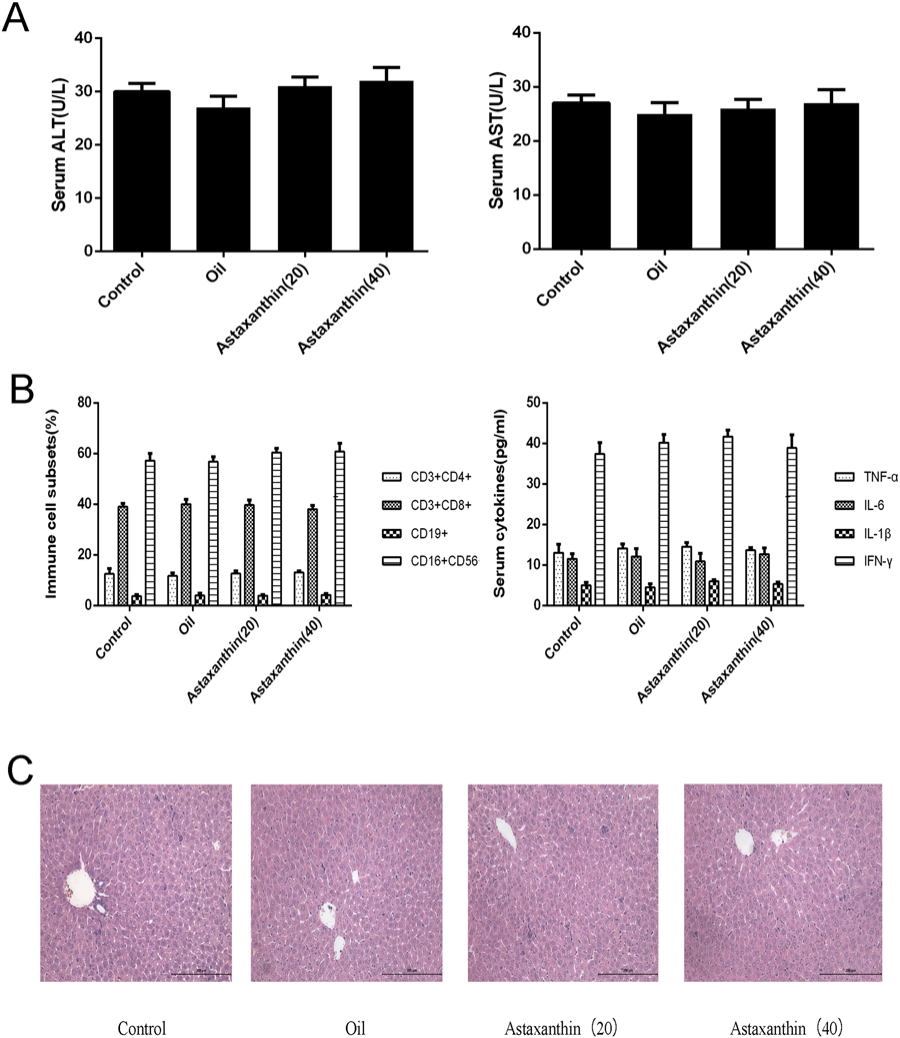
Effects of olive oil and astaxanthin on the liver function and pathology of healthy mice. (A) The levels of serum ALT and AST in the four groups did not differ. Data are given as means ± SD (n = 6, P > 0.05). (B) The percentage of different immune cell subsets, serum levels of TNF-α, IL-6, IL-1β, and IFN-γ of four groups were evaluated in each group with ELISAs or flow cytometry (n = 6, P > 0.05). (C) Representative hematoxylin-and-eosin-stained sections of the liver. Original magnification, ×200.

The drug solvent itself may affect liver function, so we analyzed the effects of olive oil and astaxanthin on liver enzymes and cytokine release. Fig. 1A shows that the levels of serum ALT, AST did not differ in the four groups, and the percentage of different immune cell subsets, serum levels of TNF-α, IL-6, IL-1β, and IFN-γ of four groups were consistent. HE staining showed no obvious necrosis in any of the slices, as shown Fig. 1C.

### 3.2 Liver injury in mice was alleviated by pretreatment with astaxanthin

**Fig 2:**
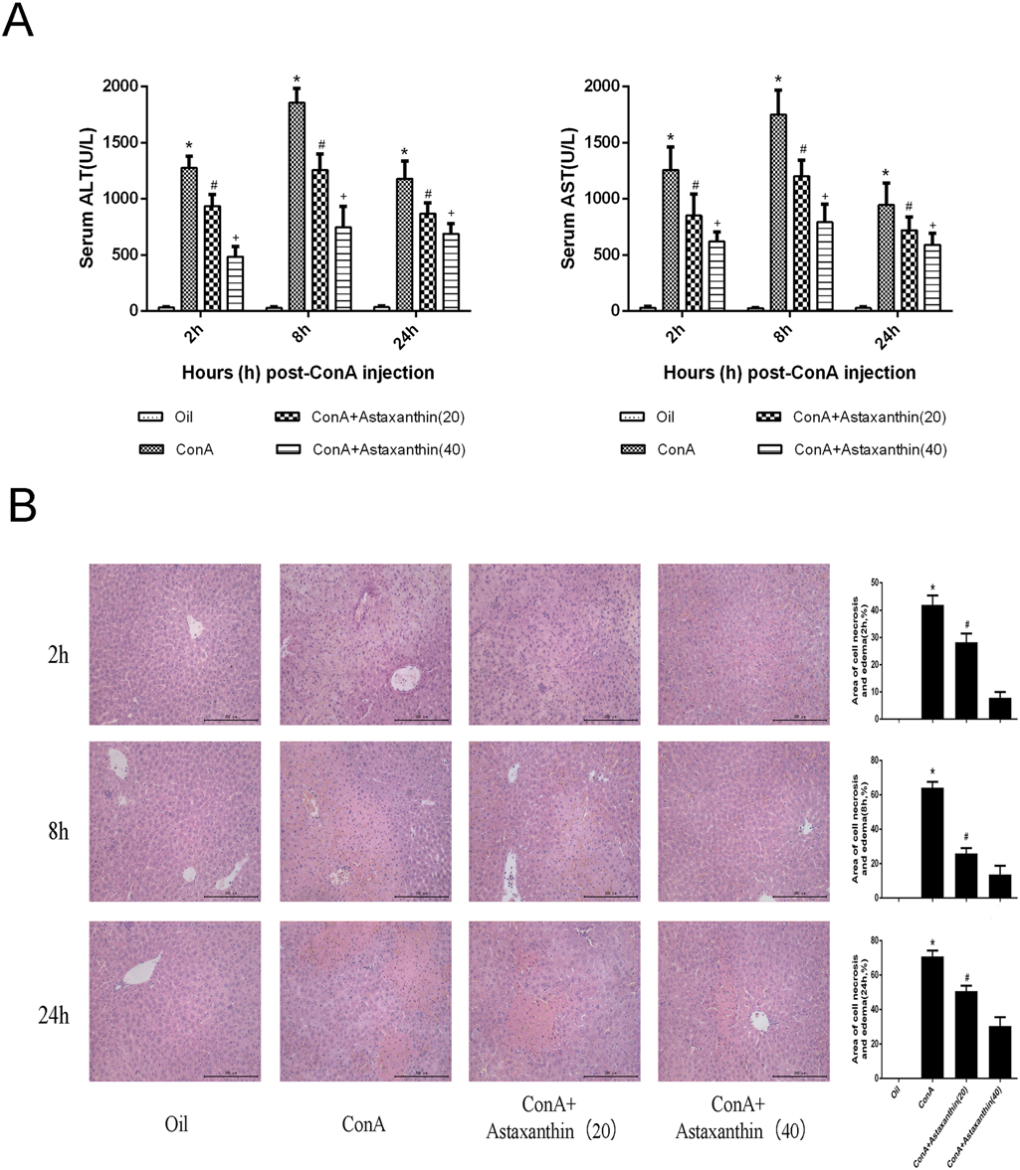
Effects of astaxanthin on liver function and pathology of mice with ConA-induced acute hepatitis. (A) The levels of serum ALT and AST changed depending on the astaxanthin dose, 20 mg/kg or 40 mg/kg. Data are given as means ± SD (n = 8, *P < 0.05 for Oil versus ConA, ^#^P < 0.05 for ConA+Astaxanthin (20) versus ConA, ^+^P < 0.05 for ConA+Astaxanthin (40) versus ConA). (B) The necrotic and edematous area stained with hematoxylin and eosin and used for the liver sections was analyzed with Image-Pro Plus 6.0 (magnification, ×200). The results show statistically significant differences among the different groups (n = 8, *P < 0.05 for ConA+Astaxanthin (20) versus ConA, ^#^P < 0.05 for ConA+Astaxanthin (40) versus ConA).

It is well established that ConA can induce immunological liver injury rapidly. Serum and liver tissue were therefore collected at 2, 8 and 24 h to evaluate the changes in liver function and necrosis. Fig. 2A shows that the levels of serum ALT and AST significantly varied among the groups at each timepoint. The most significant increase occurred in the ConA group, while astaxanthin pretreatment dramatically reduced the serum level. The high dose group showed more pronounced effects than the low dose group. Similar results were found when the necrotic and edematous area was analyzed by histopathology. Large areas of flaky necrosis and inflammatory infiltration were observed in the ConA group compared with a slight improvement in the liver tissue in the drug treatment group at 8 h. Also, the effects correlated with the dosage of astaxanthin pretreatment at every timepoint, with less necrosis evident in the high dose group, as shown in Fig. 2B. Taken together, these findings suggested that astaxanthin pretreatment can effectively improve the autoimmune liver damage caused by ConA.

### 3.3 Astaxanthin pretreatment protected the liver from the damage caused by inflammation factors

**Fig 3:**
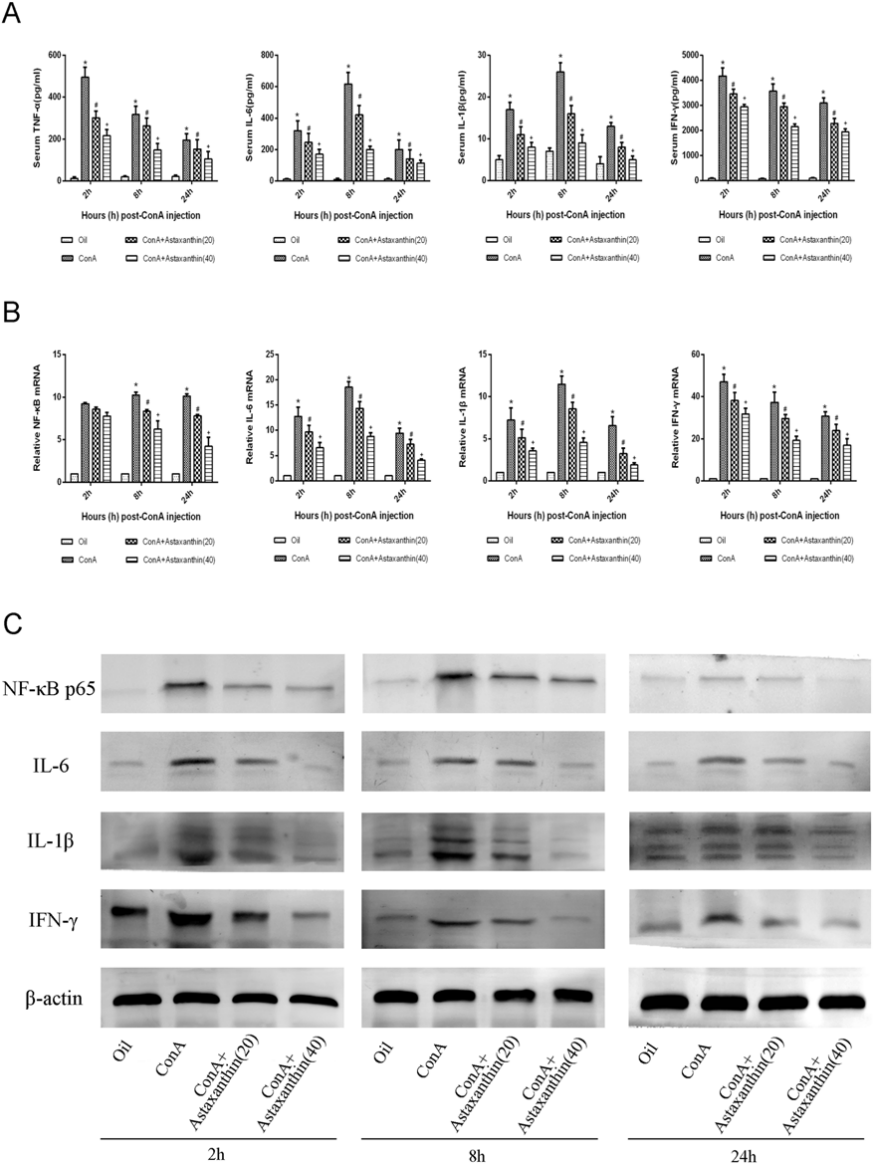
Effects of astaxanthin on the production of NF-κB p65, IL-6, IL-1β, and IFN-γ in mice with ConA-induced acute hepatitis. (A) The index of plasma TNF-α, IL-6, IL-1β, and IFN-γ, measured with ELISAs, was reduced by astaxanthin pretreatment in mice at doses of both 20 mg/kg and 40 mg/kg. Data are presented as means ± SD (n = 8, *P < 0.05 for Oil versus ConA, ^#^P < 0.05 for ConA+Astaxanthin (20) versus ConA, ^+^P < 0.05 for ConA+Astaxanthin (40) versus ConA). (B) The mRNA levels of NF-κB p65, IL-6, IL-1β, and IFN-γ were evaluated in each group with real-time PCR (n = 8, *P < 0.05 for Oil versus ConA, ^#^P < 0.05 for ConA+Astaxanthin (20) versus ConA, ^+^P < 0.05 for ConA+Astaxanthin (40) versus ConA). (C) The expression levels of the NF-κB p65, IL-6, IL-1β, and IFN-γ proteins were determined with western blotting.

The production of inflammation factors was closely related to the degree of liver injury. As shown Fig. 3A, the plasma levels of TNF-α, IL-6, IL-1β, and IFN-γ, as detected with ELISAs, increased dramatically due to liver damage after ConA induction compared with the normal group, whereas related effectors were greatly decreased with pretreatment of astaxanthin, especially at 8 h. To verify these results, we used real-time PCR to quantitate mRNA and determine the level of transcription (Fig. 3B). The expression levels of NF-κB p65, IL-6, IL-1β and IFN-γ in the high dose group were lower than in the low dose group. The results of western blot analysis showed that protein expression levels of NF-κB p65, IL-6, IL-1β and IFN-γ in liver tissue were consistent with mRNA transcription (Fig. 3C). High protein expression levels were detected in the ConA group compared with lower expression levels in the astaxanthin pretreatment group at every time point, with the most obvious differences at 8 h. The results provided strong evidence that astaxanthin could inhibit the release of inflammatory factors, such as NF-κB p65, TNF-α, IL-6, IL-1β and IFN-γ, with the levels of these factors being consistently lower in plasma, and when measuring transcription and protein expression in the astaxanthin pretreatment group.

### 3.4 Astaxanthin down-regulated autophagy and apoptosis in ConA-induced hepatitis

**Fig 4:**
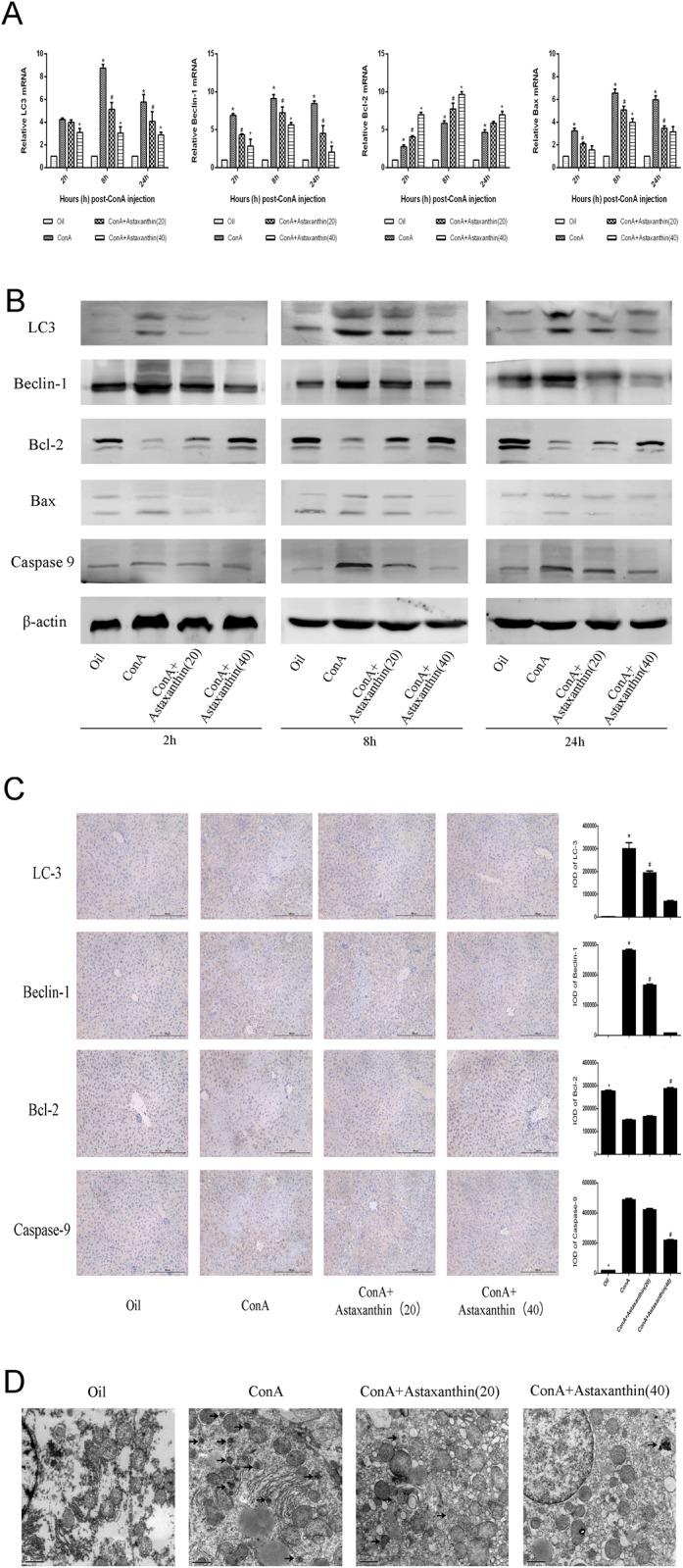
Effects of astaxanthin on apoptosis and autophagy in mice with ConA-induced acute hepatitis. (A) cDNA levels of LC3, Beclin-1, Bcl-2, and Bax were measured with real-time PCR (n = 8, *P < 0.05 for Oil versus ConA, ^#^P < 0.05 for ConA+Astaxanthin (20) versus ConA, ^+^P < 0.05 for ConA+Astaxanthin (40) versus ConA). (B) Protein expression of LC3, Beclin-1, Bcl-2, Bax, and caspase 9 was detected with western blotting. (C) Immunohistochemistry was used to detect LC3, Beclin-1, Bcl-2, and caspase 9 (original magnification, 200). The integrated optical densities (IODs) of different the indices are expressed as means ± SD (n = 8, ^+^P < 0.05 for Oil versus ConA, *P < 0.05 for ConA+Astaxanthin (20) versus ConA, ^#^P < 0.05 for ConA+Astaxanthin (40) versus ConA). (D) Autophagosome formation was detected in liver tissues with transmission electron microscopy at 8 h (magnification, 10,000). Arrows indicate autophagosomes.

LC3 and Beclin1 are important markers of autophagy, and Bax and Bcl-2 play vital roles in the regulation of apoptosis. Similarly to the inflammatory markers, real time PCR and western blot technologies were applied to assess the activation of autophagy and apoptosis at the transcriptional and protein levels, respectively, in liver tissue (Fig. 4A&4B). LC3 and Beclin1 expression decreased with increased drug dose, with the ConA treatment group presenting the highest expression levels. For the apoptotic markers, astaxanthin promoted the expression of anti-apoptosis protein Bcl-2 but inhibited the pro-apoptotic proteins Bax and caspase-9. These results were consistent with the changes in immunohistochemistry (Fig. 4C). In addition, electron microscopy was used to detect autophagosomes in liver tissue (Fig. 4D). Compared with the normal group, agglutinative chromatin, damaged mitochondria and many lysosomes and autophagosomes were identified in the ConA group. After the gavage administration of astaxanthin, the cellular injuries described above were less easily detected. Taken together, these results indicated that astaxanthin could inhibit autophagy and apoptotic processes caused by ConA to reduce pathological damage of the liver.

### 3.5 Astaxanthin attenuates JNK signal pathway by blocking the interaction between TNF-α and TRAF2

**Fig 5:**
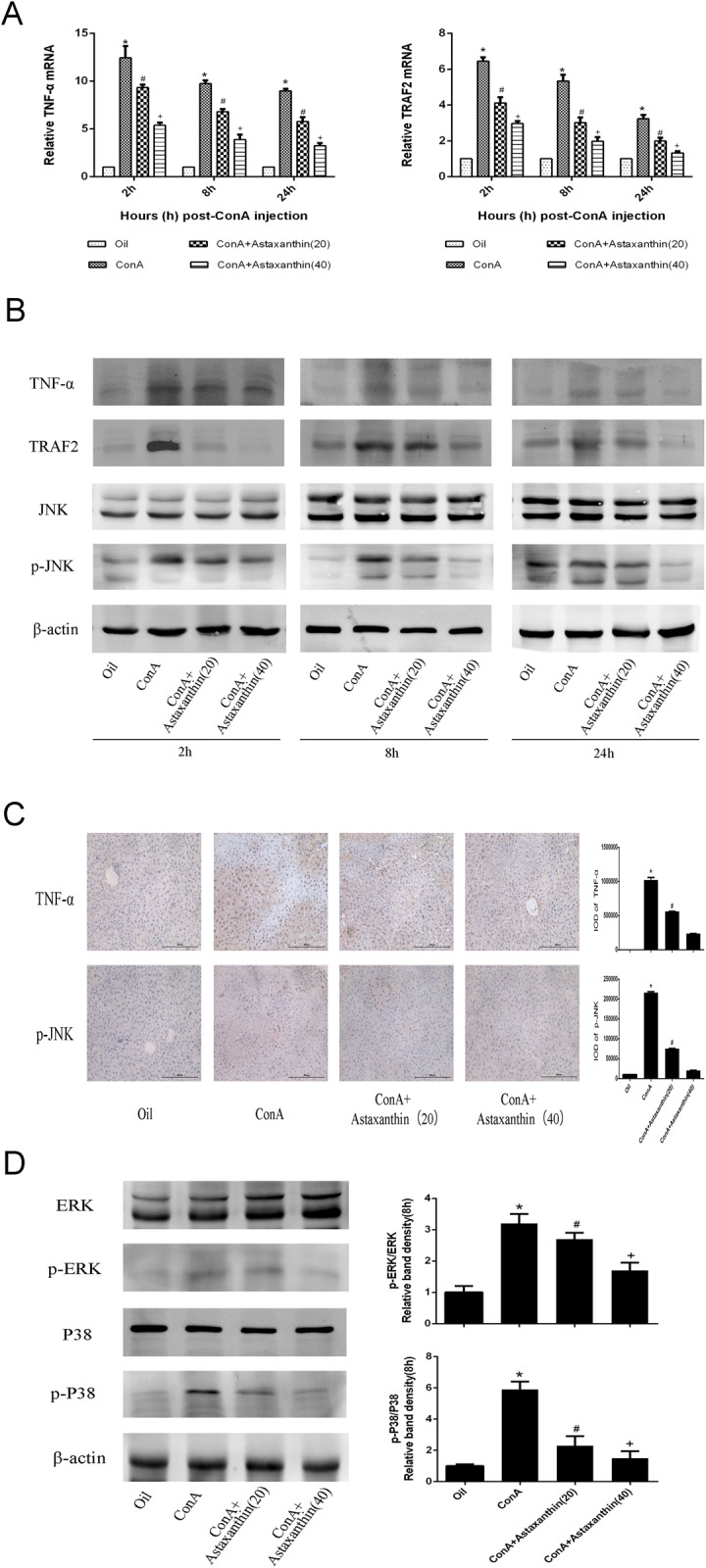
Effects of astaxanthin on the regulation of the TNF-α/JNK/p-JNK pathway in mice with ConA-induced acute hepatitis. (A) The expression of TNF-α and TRAF2 was determined with real-time PCR (n = 8, *P < 0.05 for Oil versus ConA, ^#^P < 0.05 for ConA+Astaxanthin (20) versus ConA, ^+^P < 0.05 for ConA+Astaxanthin (40) versus ConA). (B) The levels of proteins TNF-α, TRAF2, JNK, and p-JNK in liver tissue are shown as western blot bands. (C) The expression of TNF-α and p-JNK in hepatic tissues was determined with immunohistochemistry at 8 h (original magnification, 200) and their IODs changed significantly with astaxanthin treatment (n = 8, *P < 0.05 for ConA+Astaxanthin (20) versus ConA, ^#^P < 0.05 for ConA+Astaxanthin (40) versus ConA). (D) The levels of proteins ERK, p-ERK, P38 MAPK, and p-P38 MAPK in liver tissue are shown as western blot bands. The relative band densities were calculated (n = 3, *P < 0.05 for Oil versus ConA, ^#^P < 0.05 for ConA+Astaxanthin (20) versus ConA, ^+^P < 0.05 for ConA+Astaxanthin (40) versus ConA).

The JNK/p-JNK pathway has been shown to be important in up-regulating autophagy and apoptosis. To explore the mechanistic pathway of astaxanthin, we measured the concentration of the activated form of JNK, phosphorylated JNK (p-JNK), in plasma and liver tissue. As shown in Fig. 5B, ConA activated the up-regulation of JNK phosphorylation, and astaxanthin weakened this effect at all timepoints. In liver tissue and at the protein level, p-JNK expression at the high dose of astaxanthin was lower than that in the ConA group and the low dose group. The consistency between these results and immunohistochemical staining suggested that the JNK signaling pathway was attenuated by astaxanthin through inhibiting JNK phosphorylation (Fig. 5C).

Previous studies have shown that astaxanthin does not directly interact with the JNK signal pathway, and that there are many adjustment factors associated with p-JNK production. TNF-α plays an essential role in ConA-induced damage and was proposed to play a part, along with its receptor TRAF2, in the phosphorylation process of JNK. The expression of TNF-α and TRAF2 in plasma and tissue, as shown in Fig. 5, was consistent with the changes in p-JNK. These results revealed that astaxanthin downregulated the JNK signal pathway by inhibiting the combination of TNF-α and TRAF2. This trend kept pace with other members of the MAPK family, including ERK and P38 MAPK (Fig. 5D)

### 3.6 Astaxanthin protected the proliferation of primary hepatocytes induced by TNF-α and inhibited their apoptosis

**Fig 6:**
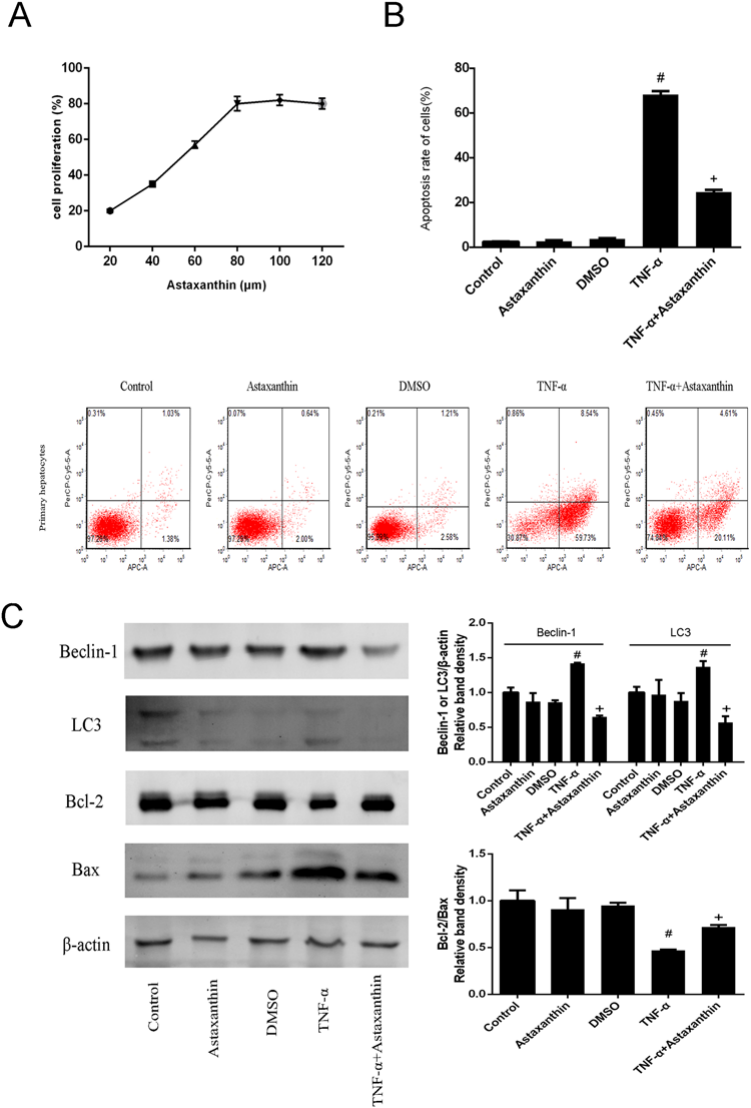
Effects of astaxanthin on the proliferation and apoptosis of primary hepatocytes induced by TNF-α. (A) The proliferation of primary hepatocytes treated with astaxanthin before TNF-α induction was detected with CCK8. (B) The apoptosis of primary hepatocytes was determined with flow cytometry (n = 3, ^#^P < 0.05 for TNF-α versus control, ^+^P < 0.05 for TNF-α+Astaxanthin versus TNF-α). (C) The protein levels of Beclin-1, LC3, Bcl-2 and Bax proteins in primary hepatocytes are shown as western blot bands. The relative band intensities were calculated (n = 3, ^#^P < 0.05 for TNF-α versus control, ^+^P < 0.05 for TNF-α+Astaxanthin versus TNF-α).

CCK8 is commonly used to measure cell proliferation. Our results show that primary hepatocytes treated with increasing concentrations (20–120 μM) of before TNF-α damage proliferated astaxanthin dose dependently (Fig. 6A). This indicates that astaxanthin protected the primary hepatocytes from inflammatory damage. We selected 80 μM as an effective dose of astaxanthin for our subsequent experiments. The results of flow cytometry and western blotting showed that the primary hepatocytes appeared a secure apoptosis after the administration of TNF-α. However, pretreatment with astaxanthin significantly reduced the percentage of apoptotic cells, as evident in the changes in Bcl-2 and Bax (Fig. 6C).

## Discussion

In recent years, the incidence of autoimmune hepatitis has increased worldwide [45]. The search for a safe and effective therapy is therefore more important than ever. Astaxanthin, a powerful antioxidant, has attracted the attention of scientists.

The ConA mouse model is a well-established model to explore liver injury caused by an inflammatory response. Recent research has shown that autoimmune hepatitis was associated with the release of large amounts of inflammatory cytokines, such as TNF-α, IL-6, IL-1β and IFN-γ, leading to apoptosis and necrosis in liver pathology [8,14]. TNF-α, secreted by the liver Kuffer cells, has been shown to play a particularly important role elevating not only the expression levels of ALT and AST but also leading to necrosis of the liver tissue [14,23]. Three separate previous studies showed that astaxanthin could perform anti-inflammatory effects through the inhibition of the NF-κB p65 or SHP-1 pathway to reduce TNF-α levels in LPS/GalN-administered mice, high-fat-fed mice, or U937 cells [33,42,46]. However, the mechanism of action of astaxanthin in immunological liver injury remained unclear.

In this study, we investigated the mechanism of action of astaxanthin in ConA-induced autoimmune hepatitis. We found that pretreatment of mice with astaxanthin could be beneficial for ConA-induced immune injury prompted by conversion of the serum liver enzyme, the release of inflammatory factors and pathological changes. The serum ALT and AST levels and the area of necrosis on biopsy exhibited a significant decline with an increasing dosage of astaxanthin (20 mg/kg versus 40 mg/kg), indicating that astaxanthin has beneficial effects on liver function in ConA-induced hepatitis. PCR and western blot analysis demonstrated a reduction in the levels of inflammatory factors IL-6, IL-1β, IFN-γ and most significantly TNF-α, after astaxanthin treatment. We considered whether astaxanthin could protect the liver from damage though inhibiting the combination between TNF-α and the membrane receptor TNFR1, causing an increase in TRAF2, TRADD and FADD, and inhibiting the JNK phosphorylation pathway [16].

Mitogen-activated protein kinases (MAPKs), a class of serine—threonine protein kinase activated by different extracellular stimuli, such as cytokines, neurotransmitters, hormones, and cell stress, can be divided into several subgroups that are all associated with TNF-α: ERK, P38, and JNK. JNK, an important branch of the MAPKs, plays an essential role in the apoptosis induced by TNF-α [47,48]. Many studies have suggested that TNF-α-induced JNK signaling is responsible for most systemic diseases [49,50]. In human dental pulp fibroblast-like cells (HPFs), the activation of cAMP response element-binding protein (CREB) via the JNK pathway in the presence of TNF-α enhanced metalloproteinase-3 production [51]. Tumor necrosis factor receptor associated factor 6 (TRAF6), upregulated in spinal cord astrocytes in the late phase of nerve injury, maintains neuropathic pain by integrating the TNF-α/JNK pathways [52]. Streetz and colleagues validated the significance of prolonged activated JNK on hepatocyte damage caused by TNF-α in ConA-induced liver injury in vivo and in vitro [53]. After ConA was injected, JNK was phosphorylated to form phosphor-JNK (p-JNK) that migrated to the mitochondrial membrane or cell nucleus causing tissue damage. Hideaki and colleagues demonstrated that the antioxidant butylated hydroxyanisole (BHA) inhibited JNK phosphorylation in mice and protected the liver tissue from ConA injury [54]. However, the mechanism of action of astaxanthin remained unclear. In this study, we used PCR, western blotting and immunohistochemical methods to demonstrate high expression of p-JNK and TRAF2 in the ConA group and low expression of p-JNK and TRAF2 in the astaxanthin-pretreatment groups. The expression of ERK and P38 MAPK proteins and their levels of phosphorylation have been shown to be consistent with JNK activation. These results suggested that astaxanthin could block the excitation of JNK through the interaction between TNF-α and TNFR1, triggering a conformational change in TRAF2. TNF-α also affects the phosphorylation of other members of the MAPK family, ERK and P38 MAPK.

**Fig 7:**
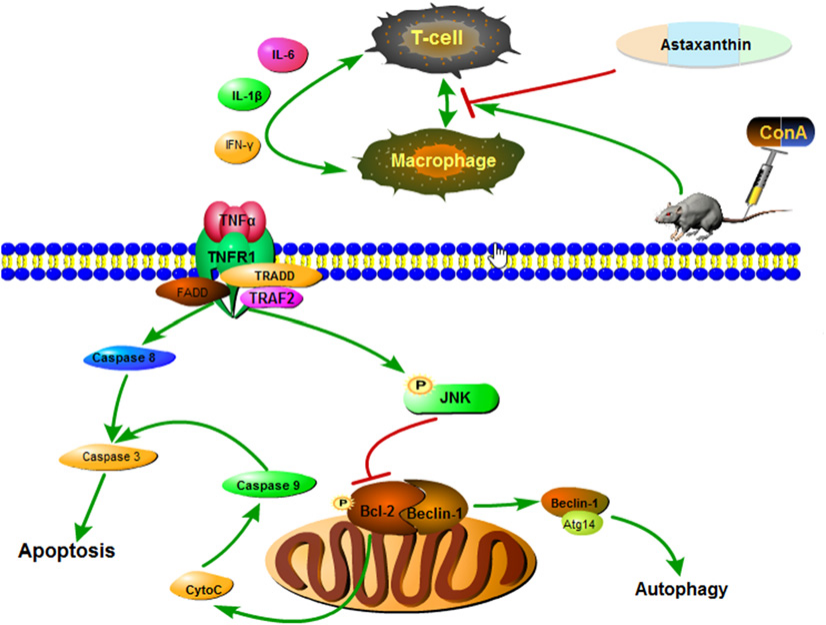
Mechanism of astaxanthin action. In ConA-induced autoimmune hepatitis, astaxanthin reduces autophagy by inhibiting the JNK/p-JNK pathway. TNF-α, a proinflammatory cytokine, combined with TNFR1 and TRAF2, was expressed on the surfaces of hepatocytes after ConA injection. This led to the activation of JNK, which phosphorylated Bcl-2, thereby promoting the release of caspase 9 and caspase 3, causing apoptosis. Inactive Bcl-2 dissociated from Beclin-1, enhancing the induction of autophagy. Thus, astaxanthin successfully inhibits the release of TNF-α in stressed cells during acute liver injury and also reduces apoptosis and autophagy by reducing the phosphorylation of JNK.

This led to the question of how astaxanthin regulates the activation of JNK to reduce liver tissue damage. The Bcl-2 family includes pro-apoptotic members (Bax, Bak and Bok) and anti-apoptotic members (Bcl-2, Bcl-xl, Bcl-2 and Mcl-1) that can mediate permeabilization of the mitochondrial membrane, a crucial element of apoptosis [55]. JNK has been shown to migrate to the mitochondrial membrane to phosphorylate and suppress Bcl-2 and Bcl-xl after its activation, thereby promoting the opening of the permeability transition pore to release cytochrome C and initiate apoptosis by caspase 9 and caspase 3 [56,57]. Our experimental results showed significant changes in the levels of apoptosis-related proteins, such as Bcl-2, Bax and caspase 9, after astaxanthin treatment through the effect of p-JNK. The increase in Bcl-2 and the reduction in Bax and caspase 9 indicate that astaxanthin inhibits the JNK/p-JNK pathway, and that the phosphorylation of Bcl-2 has an antiapoptotic effect in ConA-induced hepatitis. In recent years, research had shown that Bcl-2 can also play a role in the crosstalk between autophagy and apoptosis, mainly by the Bcl-2/Beclin-1 complex [27,55,58]. Inactive Bcl-2 induced by p-JNK phosphorylation dissociates from Beclin-1, and the free Beclin-1 enhances the induction of autophagy, as shown in Fig. 7. When forming autophagosomes, the cytoplasmic marker LC3-I is converted to membrane-type LC3-II by enzymatic hydrolysis [59]. In our mouse model of astaxanthin pretreatment, the effects of Beclin-1 and LC3-II and the dose changes at the gene and protein levels suggest that astaxanthin facilitates crosstalk between Bcl-2 and Beclin-1, and converts LC3-I to LC3-II, which reduces apoptosis and inhibits autophagy, respectively. The effective function of astaxanthin was verified in primary liver cells from mice, as shown in Fig. 6. The mechanisms involved in ConA-induced hepatitis are complex and multifactorial. The intricacies of these mechanisms are still to be fully elucidated and the protective role of astaxanthin in immune injury requires further exploration.

## Conclusions

In summary, our findings showed that astaxanthin reduces immune liver injury caused by ConA via JNK/p-JNK-mediated apoptosis and autophagy. Firstly, astaxanthin attenuated serum liver enzymes and pathological damage by inhibiting the release of inflammatory factors, such as TNF-α, IL-6, IL-1β and IFN-γ. Secondly, astaxanthin performed its anti-apoptotic effects via the descending phosphorylation of Bcl-2 activated by the TNF-α-mediated JNK/p-JNK pathway. The unseparated Bcl-2 and Beclin-1 complex failed to upregulate autophagic activity, leading to the phagocytosis of organelles and reducing liver tissue damage. Our findings highlight astaxanthin as a promising potential therapeutic agent for autoimmune hepatitis.

## Supporting Information

S1 Checklist NC3Rs ARRIVE Guidelines Checklist. The studies in vivo were in accordance with the ARRIVE guidelines, including how many mice were used, the sex of the mice, whether anesthesia and the method of sacrifice in the methods section of my manuscript.
(PDF)
